# 中国淋巴瘤患者全程管理模式专家共识（2021年版）

**DOI:** 10.3760/cma.j.issn.0253-2727.2021.05.003

**Published:** 2021-05

**Authors:** 中国临床肿瘤学会（CSCO）中国抗淋巴瘤联盟 中国医师学会血液科医师分会

淋巴瘤是一种起源于淋巴结和淋巴组织的免疫系统恶性肿瘤，其发生大多与免疫应答过程中淋巴细胞增殖分化产生的某种免疫细胞恶变有关。近年来淋巴瘤的发病率逐年增高[1]，但多数淋巴瘤患者经过规范化的治疗和管理，生存期可大大延长，甚至达到终身治愈。据报道，美国与日本淋巴瘤患者5年总生存（OS）率分别为68.1％和57.3％，而我国淋巴瘤患者5年OS率仅为38.4％[2]。我国淋巴瘤患者主要存在误诊率高、治疗及随访依从性差等问题[3]–[5]。淋巴瘤患者目前接受规范化治疗的情况并不理想，患者治疗及随访的依从性较差，有研究显示，仅22.1％弥漫大B细胞淋巴瘤（DLBCL）患者和44.8％滤泡淋巴瘤（FL）患者能够接受标准化8个及以上疗程治疗[6]。淋巴瘤兼顾慢性病和肿瘤两个特征，因此淋巴瘤患者的全程管理尤为重要[7]–[9]。我们组织了具有全程管理经验的淋巴瘤医疗和护理专家，制定了本淋巴瘤患者全程管理的专家共识，以期为中国淋巴瘤患者全程管理提供指导意见。

一、淋巴瘤患者全程管理的定义及内容

淋巴瘤患者全程管理是指通过多学科诊疗团队（MDT）[10]–[11]引导在各个科室就诊的潜在淋巴瘤患者进行淋巴瘤相关检查，实现早期诊断，经过病理确诊后，引导确诊患者就医，实现规范化诊疗和康复期随访。淋巴瘤患者全程管理包括患者就诊引导、规范化诊疗推进和康复期随访三部分[12]。

1. 就诊引导：淋巴瘤患者的就诊引导，可帮助淋巴瘤患者尽快到相关科室就诊，进而推进淋巴瘤患者早期诊断；同时加强与病理科沟通，减少疑似患者的漏检与误检[13]–[14]。主要分为三部分工作：①院内疾病科普和宣教：通过在院内可允许的公共区域内摆放淋巴瘤疾病科普资料、播放疾病科普视频等方式，提高淋巴瘤疾病认知度。②病理科的淋巴瘤患者引导：与病理科对接，引导确诊患者正确就医。③其他科室/医联体转诊患者引导：向其他相关科室宣传，引导患者正确就医。

2. 规范化诊疗：淋巴瘤患者规范化诊疗的推进主要通过缓解患者心理压力、维护患者心理健康、增强患者疾病认知与治疗的依从性，促使患者配合临床进行足疗程的规范治疗，最终改善预后[15]，主要分为五部分工作：①初诊引导：协调就诊及完善治疗前检查。②复诊提醒：在复诊前3天，推送微信公众号的就诊提示，关爱顾问同步电话联系患者，提示患者需进行下一阶段的诊疗。患者在接收到复诊提醒后，通过微信公众平台、电话等方式进行就诊预约，确保按时诊疗[16]。③诊疗管理：全程管理关爱顾问录入患者诊疗相关信息至线上患者管理平台，并定期审核[16]。④健康教育：患者就诊期间，关爱顾问每周安排时间和有需求的患者及其家属进行一对一地沟通、心理辅导；关爱顾问将每季度组织健康教育，从疾病的认知、治疗、常见问题答疑等层面进行疾病的科普教育；从医院人文宣教的层面增强患者对疾病治疗的信心。⑤协调MDT会诊：关爱顾问协调MDT对疑难病例的会诊[17]。

3. 康复期随访：康复期随访是指完成规范化治疗之后的随访[18]，随访内容包括以下三个方面：①随访提醒：关爱顾问通过患者线上管理平台等多种方式将康复期随访时间节点告知患者，提醒患者按时进行随访。②随访信息录入：关爱顾问将随访数据录入至线上管理平台[19]。③健康教育/咨询：随访期间，患者可通过多途径进行线上或线下的咨询。

二、淋巴瘤患者全程管理关爱顾问准入标准

淋巴瘤患者全程管理关爱顾问是指在“淋巴瘤患者全程管理”中起关键作用、作为“纽带”紧密连接患者及诊疗团队、全程介入患者治疗康复的医务工作者，其主要承担患者就诊引导、规范化诊疗和康复随访、诊疗管理工作，并为患者提供信息、情感与心理支持等服务，全程呵护淋巴瘤患者，为其诊疗提供全面、全程、连续照护[20]–[21]。

淋巴瘤患者全程管理关爱顾问需满足以下资质：①本科及以上学历；②护士或其他医学相关专业人员；③从事肿瘤科、血液科、淋巴瘤专科临床工作5年及以上（硕士学位者3年及以上），具有初级以上专业技术资格；④良好的沟通协调能力和责任心，有一定的康复、营养、心理、社会学知识，并且具有一定的管理、教育及科研能力等；⑤通过淋巴瘤关爱顾问培训课程，并取得合格证书[12],[22]–[23]。

三、淋巴瘤患者全程管理关爱顾问职责

1. 患者诊断、治疗、随访期的管理：主动向患者及家属提供全程就诊、治疗、转诊、随访指导，提供适时的照护；依据既定方案，通过电话或线上平台跟踪患者的治疗、随访，提醒患者按时治疗、复诊，协助患者完成规范化治疗[24]。

2. 协助患者与医护团队沟通：与诊疗团队或相关科室联络、沟通，引导患者实现规范化诊疗，必要时协调患者参加MDT会诊；与医护团队沟通，了解患者全程诊疗计划，解答患者疑问；协同临床医师评估患者病情或个人需求，协助患者转诊，必要时跟进后续治疗与随访。

3. 提供患者教育和咨询：评估患者各个阶段的生理、心理、社会需求，提供个体化心理辅导，必要时转介心理专科；评估患者和家属疾病相关知识水平[25]，提供系统的疾病诊疗护理健康教育，并定期组织开展患者科普教育活动；为患者提供疾病管理咨询。

4. 患者诊疗信息管理：将患者的基本信息、诊疗信息、疾病评估信息录入至线上管理平台，并保证信息的准确性；辅导患者使用线上患者自我管理平台；定期检查患者自我管理平台使用情况，保证患者端数据输入的真实性和准确性[26]。

5. 普及全程管理的理念：协调医院内及院际资源，在允许的公共区域摆放淋巴瘤患者全程管理宣传资料；定期对医护人员和患者进行全程管理理念的宣讲，介绍淋巴瘤患者全程管理的重要性和必要性[27]–[30]。

6. 评估全程管理工作进展：定期回顾全程管理工作进展；收集全程管理工作相关问题；采用管理工具持续进行质量改进[30]。

四、淋巴瘤患者全程管理关爱顾问培训

淋巴瘤患者全程管理关爱顾问为淋巴瘤患者全程管理的实施者，是该工作模式的核心，其角色涵盖临床护理专家、教育指导者、协调者、咨询者、研究者与管理者，集教学、研究、服务等功能于一身，有利护理多元角色的发挥[31]。为进行标准化淋巴瘤患者全程管理，需制定淋巴瘤患者全程管理规范标准培训体系，使淋巴瘤患者全程管理更具规范化、专业性，让更多淋巴瘤患者通往治愈之路[32]–[35]。

淋巴瘤患者全程管理规范标准培训包括理论培训和临床实践培训两部分，理论培训授课内容见表1。

**表1:** 淋巴瘤患者全程管理规范标准培训理论授课内容

授课内容	课时
公共知识	11
淋巴瘤全程管理关爱顾问概念及工作流程	1
淋巴瘤全程管理关爱顾问角色冲突与调试	2
淋巴瘤全程管理关爱顾问的工具	2
淋巴瘤全程管理关爱顾问的成果与评量	2
淋巴瘤全程管理关爱顾问数据分析与处理	2
医保相关政策	2
专科知识	24
淋巴瘤患者的心理护理与支持	2
淋巴瘤患者的血管通路管理	2
淋巴瘤全程管理关爱顾问实务介绍-淋巴瘤	3
淋巴瘤临床表现、病理诊断及处理原则	5
淋巴瘤常见化疗药物及化疗方案	3
淋巴瘤患者放化疗及症状管理	3
肿瘤患者的营养管理	3
淋巴瘤患者全程管理关爱顾问经验分享与交流	3

临床实践培训共3个月，包含协同专科医师门诊出诊1个月；参与病房诊疗作业1个月及跨部门培训1个月，其中跨部门培训为与疾病相关科室，如门诊、CT室、病理科、中心静脉置管（PICC）门诊、心理科、内科、放疗区、营养科等，由各科室相关专家讲解。依托MDT，采用病例分析讨论的方式，对疑难复杂病例组织教学查房[36]。培训结束后考核内容包括理论知识考试、临床实践、教学科研能力和综合素质4个方面。理论内容涵盖全程管理基础知识、静脉治疗、医保、随访、药学和疾病专科知识等。临床实践技能考核由专业组组长及护士长按照评分标准进行评价。教学科研能力考核：完成个案一份或综述一篇。综合素质考核以结业答辩的形式进行考核，包括工作态度、工作能力、工作质量、工作创新性、组织管理能力5个方面[23],[37]，采用幻灯片汇报的形式进行评分。考核合格后方可承担淋巴瘤患者全程管理关爱顾问。

五、淋巴瘤患者全程管理教育

淋巴瘤患者教育贯穿整个淋巴瘤的诊疗过程，从就诊引导期、规范诊疗期、康复随访期，通过患教手册、视频、展板、授课、病友会等多种形式[36]，分阶段进行患者教育，促进患者全面认知淋巴瘤规范诊疗的重要性，使患者得到全方位呵护，提高患者依从性，实现最大化治疗获益。

1. 就诊引导期：介绍淋巴瘤患者全程管理项目，获取知情同意；淋巴瘤诊疗流程及诊疗团队介绍；介绍淋巴瘤疾病相关知识；讲解诊断期检查项目及注意事项；评估患者心理状态，必要时进行心理疏导[38]；医保政策的解读，提供医保报销相关信息。

2. 规范诊疗期：根据诊疗方案进行药物、检查、疗程等相关注意事项指导[23]；指导患者合理选择血管通路，并提供日常自我照护信息[39]；为患者提供饮食指导；指导患者合理安排休息与活动；告知患者可能出现的相关症状及预防和应对措施；告知患者复诊相关信息，并提醒及时复诊。

3. 康复随访期：介绍康复期随访方案，强调定期随访重要性；介绍康复期随访流程及相关检查注意事项；进行康复期用药指导；提供康复期居家管理相关信息（饮食、休息与运动、症状自我管理等）[40]；回归社会指导。

淋巴瘤患者全程管理就诊流程图见图1。

**图1:**
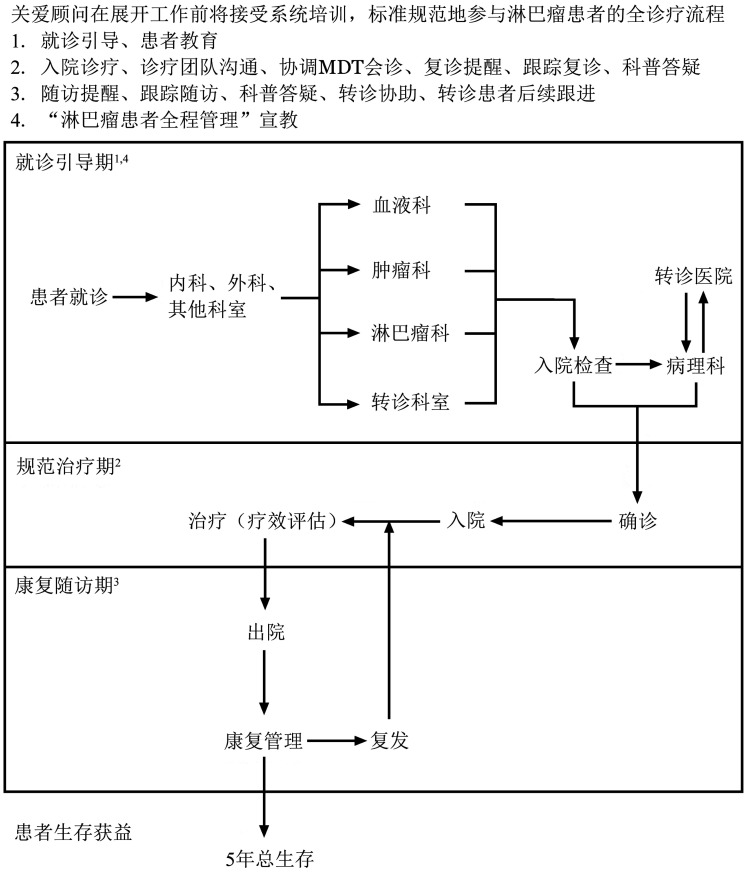
淋巴瘤患者全程管理就诊流程图

六、淋巴瘤患者全程管理质量评估

首先要建立符合医院发展的淋巴瘤患者全程管理流程，包括就诊引导流程、规范治疗流程、转介流程、随访流程等。其次设立淋巴瘤患者全程管理岗位及职责，包括编制及人力配置是否合理、岗位职责等。效果评价客观指标包括：就诊引导率、规范治疗完成率、随访率（失访率）、收案例数、5年OS率、住院天数、医疗费用、健康教育覆盖率。主观指标包括：患者满意度、患者生命质量、关爱顾问满意度[41]–[42]。

七、总结与展望

全程管理模式在我国起步较晚，目前在国内尚处于初级阶段，全程管理模式构建缺乏系统化，关爱顾问培养缺乏规范化，因此，此专家共识建立后仍需大力推动全程管理的实践，不断改善并推动专科发展，逐渐形成系统化的淋巴瘤患者全程管理模式。
